# Decentralised clinical trials for medications to reduce the risk of dementia: consensus report and guidance

**DOI:** 10.1002/alz.085781

**Published:** 2025-01-09

**Authors:** Leanne Howard, Carla Abdelnour, Erin L. Abner, Ricardo Allegri, Hiroko H Dodge, Serge Gauthier, Camilla M Hoyos, Gregory A Jicha, Patrick Gavin Kehoe, Catherine J. Mummery, Adesola Ogunniyi, Nikolaos Scarmeas, Xiaoying Chen, Jodi R. Titiner, Christopher J. Weber, Ruth Peters, The Clinical Trial, Methodology Professional Interest Area Remote Trial Working Group

**Affiliations:** ^1^ The George Institute for Global Health, University of New South Wales, Imperial College London, Sydney, NSW Australia; ^2^ Department of Neurology and Neurological Sciences Stanford University School of Medicine, Stanford, CA USA; ^3^ College of Public Health, University of Kentucky, Lexington, KY USA; ^4^ Fleni, Buenos Aires, Buenos Aires Argentina; ^5^ Harvard Medical School, Boston, MA USA; ^6^ McGill University, Montreal, QC Canada; ^7^ Macquarie University, Sydney, NSW Australia; ^8^ University of Kentucky, Lexington, KY USA; ^9^ University of Bristol, Bristol United Kingdom; ^10^ National Hospital, London United Kingdom; ^11^ University of Ibadan, Ibadan Nigeria; ^12^ Columbia University, New York, NY USA; ^13^ National and Kapodistrian University of Athens, Athens Greece; ^14^ Alzheimer Association, Chicago, IL USA; ^15^ Alzheimer’s Association, Chicago, IL USA; ^16^ University of New South Wales, Sydney, NSW Australia

## Abstract

**Background:**

Recent growth in the functionality and use of technology has prompted an increased interest in the potential for remote or decentralised clinical trials in dementia. There are many potential benefits associated with decentralised medication trials, but the field is currently lacking specific recommendations for their delivery in the dementia field.

**Method:**

A modified Delphi method engaged a panel with substantial expertise in dementia trial design and delivery and backgrounds that included neurology, psychiatry, pharmacology and psychology, to develop recommendations for the conduct of decentralised medication trials in dementia prevention. A working group of researchers and clinicians with expertise in dementia trials further refined the recommendations.

**Result:**

Overall, the recommendations support the delivery of decentralised remote trials in dementia prevention provided adequate safety checks and balances are included. A total of 40 recommendations will be presented, spanning aspects of decentralised clinical trials dementia prevention trials in phases 2b‐4, including safety, dispensing, outcome assessment, and data collection.

**Conclusion:**

With these recommendations we provide an accessible, pragmatic guide for the design and conduct of remote medication trials for the prevention of dementia.

